# Supplementary material for: Broad-Spectrum Antibiotic Use and Disease Progression in Early-Stage Melanoma Patients: A Retrospective Cohort Study
Source: Cancers (Basel). 2021 Aug 29;13(17):4367. doi: 10.3390/cancers13174367 (PMC8431240; doi:10.3390/cancers13174367)
Supplement: Supplementary file 1 [file cancers-13-04367-s001.zip › cancers-1363222-supplementary.pdf]

Supplementary table S1: Diagnosis, procedure and drug codes used in the study

**Diseases**	**ICD-9 CM and ICD-10 CM Codes**
Melanoma	"1720", "1721", "1722", "1723", "1724", "1725", "1726", "1727", "1728", "1729" (ICD-9 CM)<br><br>"C430", "C4310", "C4311", "C4312", "C4320", "C4321", "C4322", "C4330", "C4331", "C4339", "C434", "C4351", "C4352", "C4359", "C4360", "C4361", "C4362", "C4370", "C4371", "C4372", "C438", "C439", "D030", "D0310", "D0311", "D0312", "D0320", "D0321", "D0322", "D0330", "D0339", "D034", "D0351", "D0352", "D0359", "D0360", "D0361", "D0362", "D0370", "D0371", "D0372", "D038", "D039" (ICD-10 CM)
Other cancers	"140x", "141x", "142x", "143x", "144x", "145x", "146x", "147x", "148x", "149x", "150x", "151x", "152x", "153x", "154x", "155x", "156x", "157x", "158x", "159x", "160x", "161x", "162x", "163x", "164x", "165x", "166x", "167x", "168x", "169x", "170x", "171x", "174x", "175x", "176x", "177x", "178x", "179x", "180x", "181x", "182x", "183x", "184x", "185x", "186x", "187x", "188x", "189x", "190x", "191x", "192x", "193x", "194x", "195x", "200xx", "201xx", "202xx", "203xx", "204xx", "205xx", "206xx", "207xx", "208xx" (ICD-9 CM)<br><br>“C0xx”, “C1xx”, “C20x”, “C21x”, “C22x”, “C23x”, “C24x”, “C25x”, “C26x”, “C30x”, “C31x”, “C32x”, “C33x”, “C34x”, “C37x”, “C38x”, “C39x”, “C40x”, “C41x”, “C45x”, “C46x”, “C47x”, “C48x”, “C49x”, “C50x”, “C51x”, “C52x”, “C53x”, “C54x”, “C55x”, “C56x”, “C57x”, “C58x”, “C6xx”, “C71x”, “C72x”, “C73x”, “C74x”, “C75x”, “C76x”, “C81x”, “C82x”, “C83x”, “C84x”, “C85x”, “C88x”, “C90x”, “C91x”, “C92x”, “C93x”, “C94x”, “C95x”, “C96x”, “C97x” (ICD-10 CM)
Secondary malignancy (metastasis)	"196x", "197x", "198x" (ICD-9 CM)<br><br>"C77x", "C78x", "C79x" (ICD-10 CM)
Rheumatoid arthritis	"7140", "7141", "7142" (ICD-9 CM)<br><br>"M069", "M0500", "M0530", "M0560", "M061" (ICD-10 CM)

Systemic lupus erythematosus	"7100", "M3210"
Systemic sclerosis	“7101”, "M340", "M341", "M349"
HIV	"042", "043", "044", “B20”, “B21”, “B22”, “B23”, “B24”
Organ transplantation	"V420", "V421", "V422", "V426", "V427", "V428", "V429", "9968"<br><br>"T8600", "T8601", "T8602", "T8609", "T8610", "T8611", "T8612", "T8620", "T8621", "T8622", "T8640", "T8641", "T8642", "T865", "T86810", "T86811", "T86819", "T86850", "T86851", "T86859", "T86890", "T86891", "T86899", "T8690", "T8691", "T8692", "T8699", "Z940", "Z941", "Z942", "Z944", "Z9481", "Z9482", "Z9483", "Z9484", "Z9489", "Z949", "Z953"
Multiple sclerosis	“340”, “G35”
**Procedures**	**HCPCS codes**
Melanoma surgery	“11600”, “11601”, “11602”, “11603”, “11604”, “11605”, “11606”, “11610”, “11611”, “11612”, “11613”, “11614”, “11615”, “11616”, “11620”, “11621”, “11622”, “11623”, “11624”, “11625”, “11626”, “11630”, “11631”, “11632”, “11633”, “11634”, “11635”, “11636”, “11640”, “11641’, “11642”, “11643”, “11644”, “11645”, “11646”,<br><br>"17311", "17312", "17313", "17314", "17315"
Lymphadenectomy	"38740", "38745"
Sentinel lymph node excision	"38500", "38505", "38525", "38510", "38520", "38530"
Chemotherapy	Paclitaxel – "J9264", "J9267", "J9265", "C9431"<br><br>Cisplatin – "J9062", "C9418", "J9060"<br><br>Carboplatin – "J9045"

	Vinblastin – "J9360"<br><br>Carmustine – "J9050", "C9437"<br><br>Decarbazine – "J9130", "J9140"<br><br>Docetaxel – "J9170", "J9171"<br><br>Temozolomide – "J9328", "C1086", "J8700"<br><br>Granulocyte-macrophage colony-stimulating factor – "J2820"
Radiation therapy	"76873", "77014", "77261", "77262", "77263", "77280", "77285", "77290", "77293", "77295", "77300", "77301", "77305", "77310", "77315", "77321", "77326", "77327", "77328", "77331", "77332", "77333", "77334", "77336", "77338", "77370", "77371", "77372", "77373", "77401", "77417", "77427", "77431", "77432", "77435", "77470", "77520", "77522", "77523", "77525", "77750", "77761", "77762", "77763", "77776", "77777", "77778", "77785", "77786", "77787", "77789", "77790", "77799", "G0173", "G0251", "G0339", "G0340", "S2095", "G6001", "G6002", "G6003", "G6004", "G6005", "G6006", "G6007", "G6008", "G6009", "G6010", "G6011", "G6012", "G6013", "G6014", "G6015", "G6016", "G6017"
Immunotherapy	Interferon alfa-2a – "J9213"<br><br>Interferon alfa-2b – "S0146", "J9214"<br><br>Interferon alfacon-1 – "J9212"<br><br>Interferon beta-1a – "J1826"<br><br>Ipilimumab – "C9284", "J9228"<br><br>Nivolumab – "J9299"<br><br>Pembrolizumab – "J9271"<br><br>Atezolizumab – "J9022"<br><br>Durvalumab – "J9173"
Targeted therapy	National Drug Codes (NDC)

	Encorafenib – "70255002501", "702550025502"<br><br>Binimetinib - "70255001002"<br><br>Dabrafenib – "00078068166"<br><br>Trametinib – "00078066815"<br><br>Vemurafenib – "50242009002"<br><br>Cobimetinib – "50242071786"
**Antibiotics**	**Generic Product Identifier (GPI) codes**
Penicillins	"01"
Cephalosporins	"02"
Tetracyclines	"04"
Fluoroquinolones	"05"
Aminoglycosides	"07"
Sulfonamides	"08"
Carbapenems	"1615"
Oxazolidineones	"1623"
Lincomycin	"9664581010", "1622001010"
Clindamycin	"1622002010", "9646642710", "1622002022", "9005990262", "9005990219", "9005101010", "9646642730", "1622002030", "5510001810", "5510001811", "1622002031", "9005990259", "9005990359", "9005990362", "9005990265"
Vancomycin	"1600006010"
Chloramphenicol	"1620001000", "9646563648", "1620001020"

Statement—Checklist of items that should be included in reports of ***cohort studies***

	Item No	Recommendation	Page Number	Relevant text from manuscript
**Title and abstract**	1	(*a*) Indicate the study's design with a commonly used term in the title or the abstract	1, 2	Title: retrospective cohort study Methods: retrospective cohort study
		(*b*) Provide in the abstract an informative and balanced summary of what was done and what was found	1, 2	Methods: identified patients with malignant melanoma; high-dimensional propensity score approach with inverse weighting Results: final sample included; antibiotic use was associated with 31% reduction
**Introduction**				
Background/rationale	2	Explain the scientific background and rationale for the investigation being reported	3	Broad-spectrum antibiotics use also interferes with the gut flora; on the contrary, several antibiotic agents such as actinomycin and doxorubicin have antitumor activity
Objectives	3	State specific objectives, including any prespecified hypotheses	3	Our study aimed to study the association between
**Methods**				
Study design	4	Present key elements of study design early in the paper	4	Conducted a retrospective cohort study; identified patients with at least one diagnosis; excluded patients with other cancers, HIV
Setting	5	Describe the setting, locations, and relevant dates, including periods of recruitment, exposure, follow-up, and data collection	4	IQVIA PharMetrics® Plus data (January 2008–June 2018); diagnosis for malignant melanoma

				between Jan 01, 2009 and June 30, 2017
Participants	6	(*a*) Give the eligibility criteria, and the sources and methods of selection of participants. Describe methods of follow-up	4	Required these patients to have undergone either wide local excision or Mohr micrographic surgeries within 90 days of the diagnosis; excluded if they had other treatments
		(*b*) For matched studies, give matching criteria and number of exposed and unexposed		N/A
Variables	7	Clearly define all outcomes, exposures, predictors, potential confounders, and effect modifiers. Give diagnostic criteria, if applicable	7, 8	Progression in the two years after surgery was the primary outcome; exposed group was patients with prescriptions for broad-spectrum antibiotics; used pre-specified covariates; literature has shown these variables could increase the risk of melanoma; used a high-dimensional propensity score variable selection method
Data sources/ measurement	8*	For each variable of interest, give sources of data and details of methods of assessment (measurement). Describe comparability of assessment methods if there is more than one group	7, 8	Identified broad-spectrum antibiotic users using Generic Product Identifier (GPI) codes in the pharmacy files; developed an algorithm based on plausible clinical scenarios to identify progression; ICD-10 CM codes were used for identifying inpatient and outpatient diagnoses
Bias	9	Describe any efforts to address potential sources of bias	4, 8, 9	This approach was used to mitigate immortal time bias; Used inverse

				probability treatment weighting to construct pseudo populations of antibiotic users and non-users that are similar in the covariates; We examined if lack of access to healthcare could explain the difference in progression; We conducted a falsification test
Study size	10	Explain how the study size was arrived at	10, 11	Flow diagram
Quantitative variables	11	Explain how quantitative variables were handled in the analyses. If applicable, describe which groupings were chosen and why	5, 6	Table 1
Statistical methods	12	(*a*) Describe all statistical methods, including those used to control for confounding	8, 9	High-dimensional propensity score approach was used for covariate assessment; used weighted Cox proportional hazard regression for time to melanoma progression with antibiotics use as the exposure
		(*b*) Describe any methods used to examine subgroups and interactions	N/A	N/A
		(*c*) Explain how missing data were addressed	N/A	N/A
		(*d*) If applicable, explain how loss to follow-up was addressed	8	Patients' time to progression was censored for those who were not considered to be progressed
		(*e*) Describe any sensitivity analyses	9	We conducted multiple sensitivity analyses.
**Results**				
Participants	13*	(a) Report numbers of individuals at each stage of study—eg numbers potentially eligible, examined for eligibility, confirmed eligible, included in the study, completing follow-up, and analysed	9, 10	After applying the inclusion and exclusion criteria, a total of 3,930 patients remained in the sample
		(b) Give reasons for non-participation at each stage	10, 11	Flow diagram
		(c) Consider use of a flow diagram	10, 11	Flow diagram

Descriptive data	14*	(a) Give characteristics of study participants (eg demographic, clinical, social) and information on exposures and potential confounders	5, 6	Table 1
		(b) Indicate number of participants with missing data for each variable of interest	5, 6	Table 1
		(c) Summarise follow-up time (eg, average and total amount)	17, 18	Table 2
Outcome data	15*	Report numbers of outcome events or summary measures over time	17, 18	Table 2
Main results	16	(*a*) Give unadjusted estimates and, if applicable, confounder-adjusted estimates and their precision (eg, 95% confidence interval). Make clear which confounders were adjusted for and why they were included	18	Table 3
		(*b*) Report category boundaries when continuous variables were categorized	N/A	N/A
		(*c*) If relevant, consider translating estimates of relative risk into absolute risk for a meaningful time period	N/A	N/A
Other analyses	17	Report other analyses done—eg analyses of subgroups and interactions, and sensitivity analyses	20–22	Table 4
**Discussion**				
Key results	18	Summarise key results with reference to study objectives	12	was found to be associated with reduced risk of progression
Limitations	19	Discuss limitations of the study, taking into account sources of potential bias or imprecision. Discuss both direction and magnitude of any potential bias	13, 14	The study is subject to methodological limitations; could have confounded the results; this could lead to underestimation of progression; this could potentially explain, at least partially, the lower risk of progression in that group
Interpretation	20	Give a cautious overall interpretation of results considering objectives, limitations, multiplicity of analyses, results from similar studies, and other relevant evidence	14	Given the retrospective nature of the study with the data lacking clinical information on tumor characteristics
Generalisability	21	Discuss the generalisability (external validity) of the study results	14	Further studies are required to replicate and confirm the findings

**Other information**			
Funding	22	Give the source of funding and the role of the funders for the present study and, if applicable, for the original study on which the present article is based	14

*Give information separately for exposed and unexposed groups.

Note: An Explanation and Elaboration article discusses each checklist item and gives methodological background and published examples of transparent reporting. The STROBE checklist is best used in conjunction with this article (freely available on the Web sites of PLoS Medicine at http://www.plosmedicine.org/, Annals of Internal Medicine at http://www.annals.org/, and Epidemiology at http://www.epidem.com/). Information on the STROBE Initiative is available at http://www.strobe-statement.org.
